# Virologic response of treatment experienced HIV-infected Ugandan children and adolescents on NNRTI based first-line regimen, previously monitored without viral load

**DOI:** 10.1186/s12887-021-02608-0

**Published:** 2021-03-22

**Authors:** Phionah Kibalama Ssemambo, Mary Gorrethy Nalubega-Mboowa, Arthur Owora, Robert Serunjogi, Susan Kironde, Sarah Nakabuye, Francis Ssozi, Maria Nannyonga, Philippa Musoke, Linda Barlow-Mosha

**Affiliations:** 1 Makerere University-Johns Hopkins University (MU-JHU) Research Collaboration, Upper Mulago Hill Road, Mulago, P.O.BOX 23491, Kampala, Uganda; 2 Nsambya Home Care Project (NHC), Kampala, Uganda; 3 Clarke International University (Formerly IHSU), Namuwongo, Kampala, Uganda; 4 Department of Epidemiology and Biostatistics, School of Public Health, Indiana University, Indiana, USA; 5 Department of Paediatrics and Child Health, College of Health Sciences, Makerere University, Kampala, Uganda

**Keywords:** HIV, Antiretroviral therapy, Children and adolescents, Second-line, Switch, viral load, treatment failure, monitoring & response

## Abstract

**Background:**

Many HIV-infected African children gained access to antiretroviral treatment (ART) through expansion of PEPFAR programs since 2004 and introduction of “Test and Treat” WHO guidelines in 2015. As ART access increases and children transition from adolescence to adulthood, treatment failure is inevitable. Viral load (VL) monitoring in Uganda was introduced in 2016 replacing clinical monitoring. However, there’s limited data on the comparative effectiveness of these two strategies among HIV-infected children in resource-limited settings (RLS).

**Methods:**

HIV-infected Ugandan children aged 1–12 years from HIV-care programs with > 1 year of first-line ART using only immunologic and clinical criteria to monitor response to treatment were screened in 2010. Eligible children were stratified by VL ≤ 400 and > 400 copies/ml randomized to clinical and immunological (control) versus clinical, immunological and VL monitoring to determine treatment failure with follow-up at 12, 24, 36, and 48 weeks. Plasma VL was analyzed retrospectively for controls. Mixed-effects logistic regression models were used to compare the prevalence of viral suppression between study arms and identify factors associated with viral suppression.

**Results:**

At baseline all children (*n* = 142) were on NNRTI based ART (75% Nevirapine, 25% efavirenz). One third of ART-experienced children had detectable VL at baseline despite high CD4%. Median age was 6 years (interquartile range [IQR]: 5–9) and 43% were female. Overall, the odds of viral suppression were not different between study arms: (arm by week interaction, *p* = 0.63), adjusted odds ratio [aOR]: 1.07; 95%CI: 0.53, 2.17, *p* = 0.57) and did not change over time (aOR: 0 vs 24 week: 1.15; 95% CI: 0.91, 1.46, *p* = 0.24 and 0 vs 48 weeks: 1.26; 95%CI: 0.92, 1.74, *p* = 0.15). Longer duration of a child’s ART exposure was associated with lower odds of viral suppression (aOR: 0.61; 95% CI: 0.42, 0.87, *p* < .01). Only 13% (9/71) of children with virologic failure were switched to second-line ART, in spite of access to real-time VL.

**Conclusion:**

With increasing ART exposure, viral load monitoring is critical for early detection of treatment failure in RLS. Clinicians need to make timely informed decisions to switch failing children to second-line ART.

**Trial registration:**

ClinicalTrials.gov NCT04489953, 28 Jul 2020. Retrospectively registered. (https://register.clinicaltrials.gov).

## Background

The 2017 UNAIDS report estimated that 2.1 million children under the age of 15 years were living with HIV, with the majority of these living in Sub-Saharan Africa. Currently in Uganda, an estimated 47% of children living with HIV are receiving antiretroviral therapy (ART) [1]. Although ART became available about a decade ago, accessibility and coverage among treatment-eligible children and adolescents in resource-limited settings is lower than adults with 60% of eligible children receiving ART compared to 84% adults [2–6]. Efforts made towards stepping up access to first-line ART have led to positive immunological and virologic outcomes with more children maintained on ART for a longer period of time into adolescence and adulthood [7–9]. However, prolonged use of ART predisposes these children to treatment failure over time [10–13].

Monitoring response to therapy in HIV-infected children has previously been done using CD4 cell counts (immunologic) and clinical criteria because of limited access, lack of technical expertise, inadequate laboratory infrastructure and the high costs associated with viral load testing. This was linked to missed opportunities for timely identification of virologic failure and switch to second-line ART among candidate children (i.e. those who may have benefited from second-line ARV therapy), therefore paving way for emergence of drug resistance [14–17]. In Uganda, the use of immunologic criteria to determine when to switch to second-line ART had low sensitivity (13%) as a marker of viral suppression [18, 19]. Currently, WHO recommends virologic monitoring as the gold standard for diagnosing treatment failure [20, 21]. This is in anticipation of more children with poor response to ART being identified on time and given the appropriate management to improve overall survival and quality of life. In 2016, Uganda began implementing viral load testing for all pediatric patients on ART twice a year [22, 23].

Although there have been some studies done in low income countries that have highlighted the importance of measuring viral load as a more robust method of monitoring response to ART in resource limited settings [24–28], little evidence exists for the effectiveness of such a practice among treatment experienced children previously monitored without viral loads (VL), in resource limited settings. Unlike recent ART initiators, treatment-experienced children may have been maintained on a failing regimen for a considerable amount of time without documentation of virologic failure (based on clinical and immunologic information). Therefore, the objectives of this study were to (1) compare the prevalence of viral suppression and associated predictors between ART treatment-experienced children randomized to either clinical and immunological (control arm) or clinical, immunological plus VL monitoring (intervention arm) and (2) compare prevalence and correlates of decisions to switch to second-line ART between the two study arms at 12, 24, 36, and 48 weeks of follow-up.

## Methods

### Study design

We conducted a two-center, parallel-group randomized clinical trial study in Kampala, Uganda. The study adhered to CONSORT guidelines. HIV-infected children between the ages 1 and 12 years on ART were randomly assigned using a computer-generated list of random numbers (by the study statistician). Randomization was implemented in blocks (size = 4) stratified based on baseline VL (≤ 400 and > 400 HIV RNA copies/ml) to one of two parallel groups - clinical and immunological versus clinical, immunological plus virologic (VL) monitoring for treatment failure with a 1:1 allocation ratio. The allocation sequence was concealed from study staff enrolling participants to prevent selection bias. Study staff involved in the clinical care were aware of a participant’s randomly allocated group due to the nature of study intervention (i.e. monitoring treatment effectiveness) but participants were kept blinded to their group allocation to prevent the likelihood of behavior modification (e.g., adherence) due to knowledge of group assignment. An independent data safety monitoring committee reviewed unblinded data for patient safety; no interim analyses for efficacy or futility were performed.

### Eligibility/exclusion criteria

Eligible participants were HIV-infected children aged 1–12 years who (1) had a duration of at least 12 months on a first-line ART therapy and (2) were being monitored under a clinical and immunologic protocol to inform ART regimen change decisions prior to study enrollment. Exclusion was based on the use of second-line ART or PI-based regimen as first-line ART treatment, met immunologic criteria for second-line ART, active TB treatment, liver function tests (AST/ALT) > Grade 3* (5.1–10 times ULN or greater), and creatinine > Grade 3* (1.9–3.4 times ULN or greater) per DAIDS table for grading the Severity of Adult and Pediatric Adverse Events, Version 1.0, Dated December 2004, Clarification August, 2009.

### Study setting

The study took place at two HIV care centers - Makerere University-Johns Hopkins University (MU-JHU) Research Collaboration/MTCT-Plus program and Nsambya Home Care (NHC) in Kampala, Uganda.

#### Study intervention

We enrolled 142 ART-experienced HIV-infected children aged 1–12 years, from an existing treatment program that used clinical and immunologic criteria to monitor response to ART in Kampala, Uganda into the study. The 71 children randomly assigned to each study arm underwent laboratory and clinical evaluations every 12 weeks during the 48 weeks follow-up period. Plasma HIV-1 RNA levels were measured at the MU-JHU Core laboratory with the use of the standard Roche Amplicor v1.5 test kit for the children in the clinical, immunologic & virologic arm.

At screening, viral load was assessed but clinicians were blinded prior to randomization. Information on baseline characteristics of the children including; type of ART, prior PMTCT Nevirapine (NVP) exposure, length on ART, clinical and immunological status, and demography were collected. At enrollment and during scheduled follow up visits; medical history, physical examination and information on child’s growth i.e. weight and height, was also collected on standardized study source forms.

Adherence monitoring during study follow up was done using self-report by the caretaker and child where applicable, pill counts at the scheduled visits and cross-checked with pharmacy records. For participants who had adherence challenges, unannounced pill counts were conducted during home visits.

The children remained on their first-line antiretroviral regimen until they were determined to have treatment failure. Since drug resistance testing was not done in real time, the decision to switch was made based on the assignment of monitoring arm. For Arm 1 this was based on; inadequate weight gain and or increasing WHO clinical staging (new or recurrent WHO clinical stage 3 or 4 events), CD4 values falling to < 200 cells/mm^3^ or CD4 percent < 10% for a child aged between 2 and 5 years of age, CD4 count of <100cell/mm^3^ for a child aged 5 years and above. For Arm 2 this was based on; confirmed viral load > 1000 HIV RNA copies/ml; as well as clinical and immunologic criteria described above. Having 2 confirmed viral loads > 1000 HIV RNA copies/ml at least 2 weeks apart confirmed a virologic endpoint in the study. Clinicians continued participants on first line regimen if viral load is < 5000 HIV RNA copies/ml as per the 2010 WHO guidelines.

#### Statistical analysis

The distribution of study participant characteristics in the overall study sample and differences between study arms were examined to validate study randomization procedures respectively.

All data analyses were conducted based on the intent-to-treat protocol. Mixed-effects logistic regression models were used to compare the odds (prevalence) of viral suppression between the two study arms over time. Each child was modeled as a random effect nested within treatment group. Time was modeled as a categorical variable.

Changes in the odds (prevalence) of viral suppression by study arm assignment were examined by testing the significance of the study arm x week interaction term with and without adjustment for potential confounders. In the absence of statistically significant interactions (at a Type I error rate of 0.05), independent study arm, week and other child characteristic effects were examined and reported. A six-step directed acyclic graph (DAG) approach [29] was used to inform final model covariate selection to reduce the potential for and degree of bias (i.e. confounding and incorrect adjustments for mediators of intervention effects on viral suppression).

Similar procedures described above were used to compare the odds of switching a child to second- line ART regimens between the two study arms and identify correlates of ART change decisions. Missing values in predictor variables were imputed using an R package called MICE 2.22 [30]. MICE function utilizes an approach of chained equations to impute incomplete multivariate data. Final statistical model results were generated using SAS 9.4 (SAS Institute Inc., Cary, North Carolina) with the level of significance set at the 0.05 level.

## Results

### Baseline and follow-up summary characteristics

Overall, 142 children were enrolled in the study and were evenly randomized to either the clinical + immunologic (control) or clinical + immunologic + virologic (intervention) monitoring study arm. The distribution of baseline characteristics did not differ by study arm (Table 1). Descriptive statistics by study arm for viral suppression (defined by < 1000 copies/ml), time-independent, and time dependent variables (ARV change, absolute CD4, CD4%) at baseline, 12, 24, 36 and 48 weeks are also summarized in Table 1.

**Table 1:** Baseline characteristics of study participants (HIV-infected children) by randomized study arm assignment (*N* = 142)

Characteristic	Overall	Clinical & Immunologic(***N*** = 71)	Clinical, Immunologic & Virologic (***N*** = 71)	***P*** value
Study Site: n (%)				
Mulago	70 (49)	35 (49)	35 (49)	1.000^a^
Nsambya	72 (51)	36 (51)	36 (51)	
Age in years: Median (IQR)	6 (5, 9)	6 (5, 8)	7 (5, 9)	0.269^b^
Sex: n (%)				0.865^a^
Female	61 (43)	31 (43)	30 (42)	
Male	81 (57)	40 (56)	41 (58)	
WHO Stage: n (%)				0.947^c^
1	22 (15)	12 (17)	10 (14)	
2	57 (40)	29 (41)	28 (39)	
3	54 (38)	26 (37)	28 (39)	
4	9 (6)	4 (6)	5 (7)	
NVP Exposure: n (%)				0.938^c^
Yes	53 (37)	27 (38)	26 (37)	
No	76 (54)	37 (52)	39 (55)	
Unknown	13 (9)	7 (10)	6 (8)	
In utero ART: n (%)				0.960^c^
Yes	43 (30)	21 (30)	22 (31)	
No	82 (58)	41 (58)	41 (58)	
Unknown	17 (12)	9 (12)	8 (11)	
HIV RNA copies/ml: n (%)				0.722^a^
≤ 1000	94 (66)	46 (65)	48 (68)	
> 1000	48 (34)	25 (35)	23 (32)	
HIV RNA copies/ml: n (%)				1.000 ^a^
≤ 400	92 (65)	46 (65)	46 (65)	
> 400	50 (35)	25 (35)	25 (35)	
ART Duration in years: Median (IQR)	4 (2, 5)	3 (2, 4)	4 (2, 5)	0.225^b^
CD cell count (%): Median (IQR)	32 (26,38)	33 (26, 38)	32 (27, 38)	0.931 ^b^
Absolute CD4 cell count: Median (IQR)	1071 (759, 1416)	1097 (776, 1537)	1032 (721, 1326)	0.501 ^b^
BMI-for-age Z: Median (IQR)	−0.26 (−0.92, 0.36)	−0.21 (−1.06, 0.43)	−0.28 (−0.88, 0.29)	0.875 ^b^
Weight-for-age Z score: Median (IQR)	− 0.97 (− 1.54, − 0.25)	− 0.97 (− 1.37, − 0.33)	− 0.99 (− 1.63, − 0.15)	0.933 ^b^
Height-for-age Z score: Median (IQR)	−1.07 (− 1.92, − 0.34)	−1.10 (− 1.76, − 0.28)	−1.03 (− 1.93, − 0.41)	0.979 ^b^

Missing: %CD4–15; Abs CD4–2

^a^Chi-square test; ^b^Wilcoxon Two-Sample test; ^c^Fishers-Exact test

Of the 142 participants, 129 (91%) and 134 (94%) completed the 24- and 48-week assessments, respectively. The intervention study arm (clinical + immunologic + virologic monitoring) had additional assessments at 12 (response rate: 100%) and 36 (response rate: 96%) weeks; however, overall, there were no differences in response rate by study arm (*p* = 0.98).

### Viral suppression

**Fig. 1:**
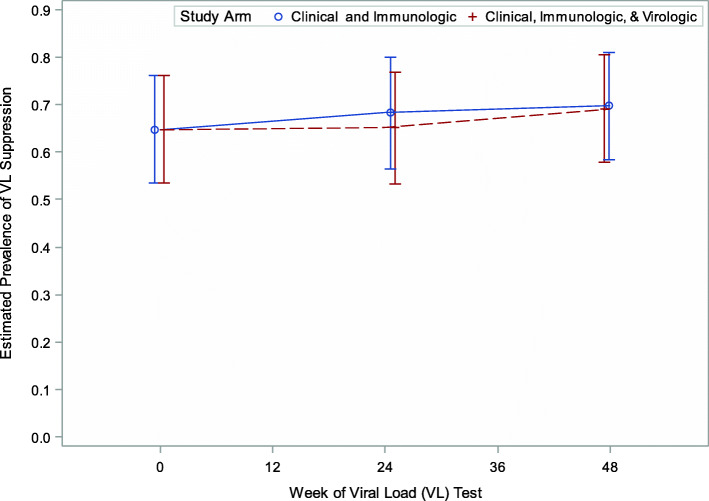
Estimated prevalence of viral load suppression (< 400 copies/ml) by study arm during a 48-week follow-up

**Fig. 2:**
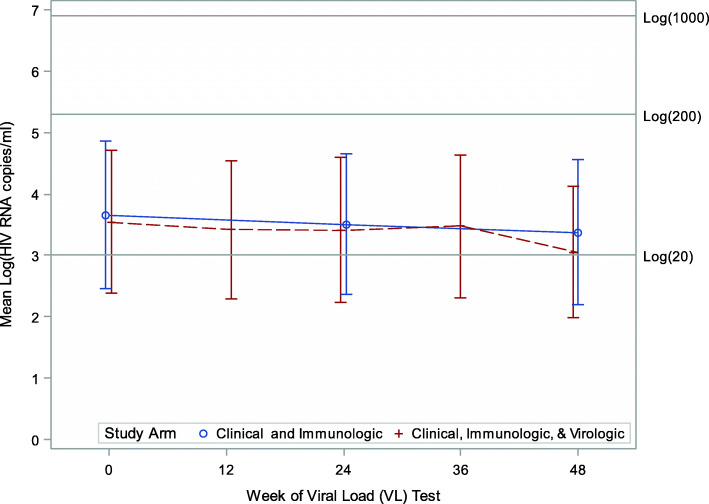
Mean Log (HIV RNA copies/ml) by study arm during a 48-week follow-up. Undetected virus levels rounded off to 1 copy/ml (log (1) =0). Overall and within-study arm median HIV RNA copies/ml was zero

**Fig. 3:**
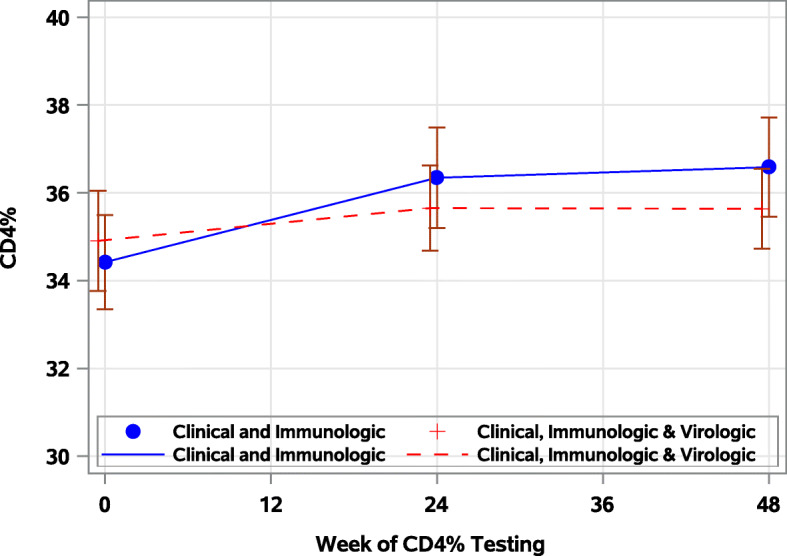
Mean CD4 cell % by study arm during a 48-week follow-up

Overall, 92 (64%), 86 (67%) and 93 (69%) children had viral suppression at baseline, 24 and 48 weeks respectively. The prevalence of viral suppression did not differ by study arm during follow-up (study arm x week interaction term: *p* = 0.63). Overall, the odds of viral suppression were not different between study arms (Clinical, Immunologic & Virologic) vs Clinical & Immunologic (Fig. 1): [aOR]: 1.07; 95%CI: 0.53, 2.17, *p* = 0.57) and did not change over time [Fig. 2] (aOR: 0 vs 24 week: 1.15; 95% CI: 0.91, 1.46, *p* = 0.24 and 0 vs 48 weeks: 1.26; 95%CI: 0.92, 1.74, *p* = 0.15). Having no history of NVP exposure (aOR: 2.75; 95%CI: 1.19, 6.37), higher baseline CD4% cell count (Fig. 3) (aOR: 1.09; 95%CI: 1.04, 1.15) or absolute (log transformed) CD4 count (aOR: 2.75; 95%CI: 1.17, 15.18) were associated with higher odds of viral suppression over time. Longer durations on ART was associated with lower odds of viral suppression (aOR: 0.67; 95%CI: 0.49, 0.91). Although, the prevalence of viral suppression was higher among children who had had a drug switch in their ARV regimen (80%) versus those that did not (66%) at 24 weeks (Table 2), overall, a change in ARV regimen (defined as 3 drug change to change to second line) did not predict viral suppression over time (aOR: 0.85; 95% CI: 0.27, 2.73). Study site, age, sex, baseline WHO stage, in-utero ART exposure, age-adjusted BMI, weight and height were not associated with viral suppression over time (Table 2).

**Table 2:** Prevalence of viral suppression, crude and adjusted odds ratios (with 95%CI) summarizing the relationship between selected risk factors and viral suppression during a 48 week follow-up period among HIV-infected children in Uganda (*N* = 142)

Characteristic	Viral suppression by week			Crude Odds Ratio	Adjusted Odds Ratio
	Baseline n (%)	24 Weeks n (%)	48 Weeks n (%)	OR (95% CI)	aOR (95% CI)
Monitoring arm					
Immunologic	46 (65)	43 (68)	46 (70)	Ref	Ref ^a^
Immunologic & Virologic	46 (65)	43 (65)	47 (69)	0.88 (0.46; 1.69)	1.06 (0.47; 2.42)
ARV Change^∫^					
No		78 (66)	77 (69)	Ref	Ref
Yes		8 (80)	16 (70)	1.52 (0.59; 3.89)	0.85 (0.27; 2.73)
Study Site					
Mulago	43 (61)	38 (59)	43 (63)	Ref	Ref ^b^
Nsambya	49 (68)	48 (74)	50 (76)	1.56 (0.82; 2.99)	0.70 (0.25; 1.96)
Sex					
Female	39 (64)	34 (63)	41 (69)	Ref	Ref ^c^
Male	53 (65)	52 (69)	52 (69)	1.12 (0.58; 2.15)	1.01 (0.45; 2.26)
WHO Stage					
1	16 (73)	12 (63)	14 (66)	Ref	Ref ^d^
2	38 (67)	40 (78)	40 (74)	1.14 (0.42; 3.09)	0.63 (0.17; 2.26)
3	33 (61)	31 (61)	33 (66)	0.74 (0.28; 1.98)	0.49 (0.14; 1.66)
4	5 (56)	3 (38)	6 (67)	0.55 (0.13; 2.32)	0.28 (0.05; 1.63)
NVP Exposure					
Yes	27 (51)	28 (55)	28 (56)	Ref	Ref ^e^
No	56 (74)	47 (72)	55 (77)	2.61 (1.31; 5.21)^**^	2.75 (1.19; 6.37)^**^
Unknown	9 (69)	11 (85)	10 (77)	2.92 (0.83; 10.32)	
In utero ART					
Yes	27 (63)	25 (60)	24 (57)	Ref	Ref ^f^
No	51 (62)	47 (65)	54 (72)	1.37 (0.67; 2.77)	1.20 (0.53; 2.74)
Unknown	14 (82)	24 (93)	15 (88)	5.10 (1.26; 20.61)^*^	–
Age (years)				1.01 (0.90; 1.16)	1.19 (0.97; 1.48)^g^
ART duration (years)				0.70 (0.56; 0.88)^**^	0.67 (0.49; 0.91)^h*^
CD%/cell count				1.03 (0.99; 1.07)	1.09 (1.04; 1.15)^i*^
Log (Absolute CD4)				2.82 (1.39, 5.69)^**^	2.75 (1.17; 15.18)^i*^
BMI-for-age Z:				0.79 (0.61; 1.01)	0.85 (0.62; 1.15)^j^
Weight-for-age Z:				0.96 (0.73; 1.28)	1.11 (0.75; 1.64)^j^
Height-for-age Z:				1.21 (0.91; 1.61)	1.33 (0.91; 1.93)^j^

*p < 0.05; **p < 0.01

^a^Adjusted for week, location, sex, age, NVP exposure, In-utero ART, ART duration, Absolute CD4

^b^Adjusted for study arm, week, sex, age, NVP exposure, In-utero ART, ART duration, Absolute CD4

^c^Adjusted for study arm, week, Absolute CD4

^d^Adjusted for study arm, week, location, sex, and in-utero ART

^e^Adjusted for study arm, week, location, WHO stage, in-utero ART, ART duration and BMI-for-age Z

^f^Adjusted for study arm, week, and location

^g^Adjusted for study arm, week, location, sex, NVP exposure, In-utero ART, and BMI-for-age Z

^h^Adjusted for study arm, week, location, WHO stage, NVP exposure, In-utero ART, and BMI-for-age Z

^i^Adjusted for study arm, week, location, sex, NVP exposure, In-utero ART, and BMI-for-age Z

^j^Adjusted for study arm, week, location, sex, NVP exposure, In-utero ART, ART duration and Absolute CD4

^∫^ARV Change – Time dependent (varying) variable (adjusted for study arm, week, location, NVP exposure, ART duration and baseline absolute CD4 count)

### ARV changes

**Fig. 4:**
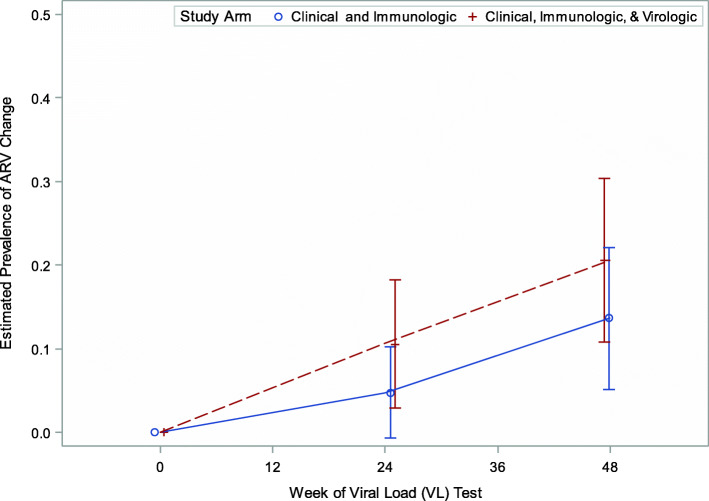
Estimated prevalence of ARV change by study arm during a 48-week follow-up, Phase I

The prevalence of ARV switching during follow-up did not differ by study arm [Fig. 4] (study arm x week interaction term = 0.39). Overall, the odds of ARV changes did not differ between study arms ([aOR]: 1.58; 95%CI: 0.62; 4.00) but increased over time; the odds of switching a child’s ARV regimen were 5.37 (aOR: 95%CI: 3.02, 7.97) and 24.10 (aOR: 95%CI: 9.14, 63.56) times higher at 24 and 48 weeks, respectively adjusted for study arm assignment and suspected confounders i.e. (log (HIV RNA copies/ml), study arm, week, NVP exposure, ART duration, and location).

The prevalence of switching to second-line ARV regimens increased over time: 8 and 17% at 24 and 48 weeks, respectively. The overall odds of switching a child’s ARV did not differ by NVP exposure, ART duration and study site (Table 3). Changes in viral load and CD4 cell count did not predict switches in ARV regimens either (Table 3).

**Table 3:** Prevalence of ARV change, crude and adjusted odds ratios (with 95%CI) summarizing the relationship between selected risk factors and ARV change during a 48 week follow-up period among HIV-infected children in Uganda (*N* = 142)

Characteristic	ARV changed by week				Crude Odds Ratio	Adjusted Odds Ratio
	12 n (%)	24 n (%)	36 n (%)	48 n (%)	OR (95% CI)	OR (95% CI)
Overall	2 (1)	10 (8)	12 (9)	23 (17)		
Monitoring arm						
Immunologic	–	3 (5)	–	9 (14)	Ref	Ref
Virologic	2 (3)	7 (11)	12 (18)	14 (21)	1.52 (0.59; 3.89)	1.58 (0.62; 4.00) ^a^
^∫^log (HIV RNA copies/ml)					0.97 (0.90; 1.05)	1.02 (0.92; 1.12)^a^
^∫^Log (ABS CD4)					0.91 (0.44; 1.86)	0.77 (0.29; 2.00)^b^
^∫^CD4%					0.99 (0.96; 1.04)	0.97 (0.92; 1.04)^b^
Week						
Baseline					Ref	Ref^*^
24					1.91 (1.19; 3.07)	5.37 (3.02; 7.97) ^a^
48					3.65 (1.40; 9.45)	24.10 (9.14; 63.56) ^a^

*p < 0.05; **p < 0.01

Participants who had an ARV change are counted in the prevalence estimate at subsequent follow-up

^a^ Covariates in the final adjusted model: log (HIV RNA copies/ml), study arm, week, NVP exposure, ART duration, and location

^b^ Covariates in the final adjusted model: study arm, week, NVP exposure, ART duration, and location

^∫^Time dependent variable

## Discussion

Recommendation for ART monitoring has gradually changed over time. Initially, patients’ CD4 T Cells were monitored every 6 months and viral load testing was only done when possible. Currently, WHO recommends viral load as the preferred monitoring approach to diagnose and confirm treatment failure, with viral load testing being conducted at 6 and 12 months after ART initiation and every 12 months thereafter [31]. Viral load monitoring in children is required every 6 months.

In this analysis of Ugandan children and adolescents, our findings show that the addition of periodic (annual) virologic testing to standard of care (clinical and immunological monitoring) among treatment-experienced HIV infected children was not associated with a higher prevalence of viral suppression. The results do however support the need for virologic monitoring as a better strategy for early detection of virologic failure especially with increased duration of ART exposure, since immunologic monitoring is a poor predictor of treatment failure. Our findings are in agreement with previous Sub-Saharan Africa studies which have shown that clinical and immunologic monitoring is not sufficient to detect virologic failure in a timely manner [14, 16, 32], which further supports the need for viral load testing as a strategy to monitor response to treatment in children. This is similar to findings from a study done in Uganda and Zimbabwe, the ARROW TRIAL which highlighted the importance of confirming virological failure before switching to second-line ART, since some children with detectable low-level viraemia spontaneously re-suppressed [33].

Additionally, our study showed no evidence to suggest virologic testing results in better treatment decisions (i.e. switching to second-line ARVs) linked to better clinical outcomes (i.e. viral suppression). Although the odds of switching a child’s ARV regimen were not different between study arms, the odds of switching to second-line regimens increased over time but were not predicted by a child’s treatment exposure history or immunological parameters. Our study highlights the need to operationalize monitoring results into algorithms to inform ARV change decisions since there appears to be a disconnect between monitoring results and actual decisions to switch ARVs.

Independent of study arm assignment, having no history of NVP exposure, higher baseline CD4% cell count or absolute CD4 count were associated with higher odds of viral suppression over time while longer duration on ART was associated with lower odds of viral suppression. A similar study done in Tanzania in perinatally HIV infected children [34] also showed that longer duration on ART was associated with poor virologic outcomes due to selection of resistance mutations making their virus more difficult to suppress. This NNRTI resistance pattern is very common in many countries, Uganda inclusive where non-nucleoside reverse transcriptase inhibitors (NNRTIs) of which nevirapine is part were used as part of a first-line ART regimen. In addition, children in this study were exposed to single dose nevirapine for PMTCT and their mothers also received nevirapine which could have lowered their odds of virologic suppression, a trend consistent with previous studies [35–38]; this may explain our null intervention effect [39, 40].

In contrast to previous studies [41, 42], our study findings suggest that the addition of periodic (annual) virologic testing did not change the prevalence of ART switching decisions between the two study arms, although the viral load monitoring was able to identify virological failure sooner than the ones who were monitored clinically and immunologically. The delay in switching could have been due to lack of clear guidelines at the time on when to switch, limited ART options and lack of confidence on the part of the health care providers to switch ART. The number of children switched to second-line ART was small in this study and if only immunological monitoring was used to determine when to switch to second-line ART, the percentage of children switched to second-line ART would have been much less. Viral load monitoring should be utilized to identify treatment failure early and thereafter make the necessary changes (28) which would reduce accumulation of drug resistance mutations and limited treatment options for second-line ART (29).

The strengths of this study include the longitudinal laboratory and clinical monitoring as well as the high rates of follow up with minimal losses to follow up during the 48-week period. The children in the two arms were comparable in terms of baseline characteristics and virologic suppression at baseline.

The limitations of this study include the partial blinding, the short follow up period of 48 weeks and a relatively small sample size of perinatally HIV infected children and adolescents, which may not be generalizable to all ART experienced children. Both the study staff enrolling participants and the participants were blinded to the assignment however, the clinical care staff were not. This could have influenced the behaviour of study staff towards participants. Longer duration of follow up and a larger sample size would confirm the findings of this study. However, the current guidelines and practice recommend that all children on ART should have routine VL monitoring.

## Conclusion

The results of this study confirm that virologic monitoring is a superior strategy for  detecting early HIV treatment failure especially with the increasing duration of ART exposure among adolescents and lend credence and support to the current WHO guidelines on ART monitoring. The issue of availability and cost of viral load testing still remains a challenge, especially in high burden countries in Sub-Saharan Africa. Therefore, studies to evaluate the affordability and accessibility of these services in resource limited settings will further inform the current WHO recommendations. In addition, there is need to operationalize monitoring results into algorithms to inform ART change decisions since there appears to be a disconnect between monitoring results and actual decisions to switch ART by health workers after ART treatment failure is identified.

## Data Availability

All data generated or analyzed during the current study are included in this published article and its supplementary information files. Additionally, the datasets analyzed during the current study may be availed from the corresponding author upon reasonable request.

## Abbreviations

ART: Antiretroviral treatment
VL: Viral load
IQR: Interquartile range
aOR: Adjusted odds ratio
RLS: Resource limited settings
